# Patient-specific instrument-assisted minimally invasive internal fixation of calcaneal fracture for rapid and accurate execution of a preoperative plan: A retrospective study

**DOI:** 10.1186/s12891-020-03439-3

**Published:** 2020-06-27

**Authors:** Chenggong Wang, Can Xu, Mingqing Li, Hui Li, Han Xiao, Da Zhong, Hua Liu

**Affiliations:** 1 Department of Orthopedics, Xiangya Hospital Central South University, No.87 Xiangya Road, Changsha, 410008 Hunan China; 2 Department of foot and ankle surgery, Xiangya Hospital Central South University, No.87 Xiangya Road, Changsha, 410008 Hunan China; 3 Department of Sports Medicine, Xiangya Hospital Central South University, No.87 Xiangya Road, Changsha, 410008 Hunan China

**Keywords:** Digital surgical simulation, Patient-specific instrument, Calcaneal fracture, Minimally invasive internal fixation, Whole process-assisted surgical technique

## Abstract

**Background:**

Traditional methods for minimally invasive internal fixation (MIIF) of calcaneal fractures require extensive intraoperative fluoroscopy, and fracture recovery is usually not ideal. We developed a new surgical procedure using digital surgical simulation and constructed a patient-specific instrument (PSI) for calcaneal fracture that we used during the operation. This study investigated whether PSI-assisted MIIF of calcaneal fracture enables rapid and accurate execution of the preoperative plan.

**Methods:**

We retrospectively analyzed patients with Sanders type III or IV fresh calcaneal fractures who had undergone PSI-assisted MIIF at our hospital from January 2016 to December 2018. We analyzed perioperative data including intraoperative fluoroscopy time, concurrence of internal fixation actual usage (IFAU) with the preoperative plan, surgery time, and complications. We also compared pre- and postoperative actual measurements from X-ray radiographs and computed tomography images including Böhler, Gissane, and calcaneus valgus angles; subtalar joint width; and calcaneal volume overlap ratio with the preoperative design. All patients had been followed up and their American Orthopedic Foot and Ankle Score (AOFAS) score was available.

**Results:**

Mean intraoperative fluoroscopy time was 3.95 ± 1.78 h; IFAU in 16 patients (16 ft) was the same as the preoperative plan; mean surgery time was 28.16 ± 10.70 min; and none of the patients developed complications. Böhler, Gissane, and calcaneus valgus angles and subtalar joint width did not differ between pre- and postoperative plans; however, the actual preoperative values of each of these parameters differed significantly from those measured postoperatively. The calcaneal volume overlap ratio with the preoperative design was 91.2% ± 2.3%. AOFAS scores increased with time, with significant differences in the score at each time point.

**Conclusions:**

The newly developed PSI-assisted calcaneal fracture MIIF method can rapidly and accurately execute the preoperative plan.

## Background

Many doctors prefer to use minimally invasive surgery to treat freshly closed calcaneal fracture [1, 2]. In this procedure, the surgeon often performs closed reduction of the fracture or open reduction with a small incision, in conjunction with percutaneous fixation [3]. This has many advantages compared to open reduction internal fixation (ORIF) [4, 5] including fewer incision-related complications [6], shorter duration of limb swelling [7], shorter recovery time [8], and lower rate of fixation failure [9].

The classic minimally invasive surgical method for treating calcaneal fractures involves fracture reduction, temporary fixation, and internal fixation with an implant. However, this method has certain disadvantages. Firstly, the procedure requires extensive intraoperative fluoroscopy to observe the fracture and repeatedly examine internal fixation at each step of the operation [10]. Secondly, fracture recovery and internal fixation are frequently found to be less than ideal at follow-up, because the success of the procedure depends on the surgeon’s experience [11].

Digital surgical simulation can optimize the surgery process by increasing precision and personalization [12]. 3-Dimensional (3D) printing technology and patient-specific instruments (PSIs) have provided useful tools for orthopedic surgery [13]. Based on years of experience examining calcaneus biomechanics and using digital orthopedic technologies such as PSI-assisted surgery [14–16], we developed an improved method of classical calcaneal fracture minimally invasive internal fixation (MIIF) by shifting the focus of surgery from internal conditions to external auxiliary tools. In order to achieve accurate anatomic reduction of calcaneal fractures in MIIF and prevent the occurrence of traumatic arthritis of the subtalar joint, we designed the new surgical procedure through digital surgical simulation and constructed a PSI for calcaneal fracture surgery. This allowed step-by-step execution of the preoperative plan, in contrast to traditional surgery methods. The new technique has been validated in numerous trials including a clinical study at our hospital, and has been approved by ethics committees. In the present retrospective study, we investigated whether PSI-assisted calcaneal fracture MIIF can rapidly and accurately execute the preoperative plan.

## Methods

### Patients

This study was approved by the Ethics Committee of Xiangya Hospital. We retrospectively analyzed data from patients with Sanders type III or IV fresh calcaneal fractures who had undergone calcaneal fracture MIIF at our hospital from January 2016 to December 2018. Data were obtained from the registration system of the Foot and Ankle Surgery Department, medical record information system, and patient service center follow-up system at our hospital. All patients included in this retrospective study provided written, informed consent for their participation. All operations were carried out by one of the authors (H.L.), who has performed over 150 calcaneal fracture internal fixation surgeries. The inclusion criteria were: 1) fresh calcaneal fracture (within 72 h of injury); 2) Sanders type III or IV fracture; 3) treated by PSI-assisted surgery; and 4) voluntarily provided medical records for this study. The exclusion criteria were as follows: 1) refused to participate in the study (6 patients); 2) other operations performed on the homolateral lower limb (1 patient); 3) physical activity disorder caused by disease (e.g., stroke) (2 patients); 4) preoperative preparation time too short to prepare the PSI (9 patients); and 5) mental illness (1 patient). In total, 19 patients (20 ft) were enrolled in our study and were followed up. The cohort included 11 males and 8 females with the average age of 37.90 ± 13.63 years (range: 19–65 years). Patients were diagnosed with Sanders III or IV fresh calcaneal fracture by an experienced orthopedic surgeon based on standard computed tomography (CT) scanning. Seven feet were diagnosed as Sanders III and 13 as Sanders IV; 12 patients were diagnosed with left calcaneal fracture, 6 with right calcaneal fracture, and 1 with bilateral fractures. All subjects were examined by X-ray radiography and CT before their operation (Table 1).

**Table 1:** Demographic characteristics of calcaneal fracture patients in this study

Characteristic	Number(***N*** = 19)
Sex (male/female)	11/8
Mean age (years)	37.90 ± 13.63
Side (left/right/bilateral)	12/6/1
Sanders classification (III/IV)	7/13

### Preoperative planning

**Fig. 1:**
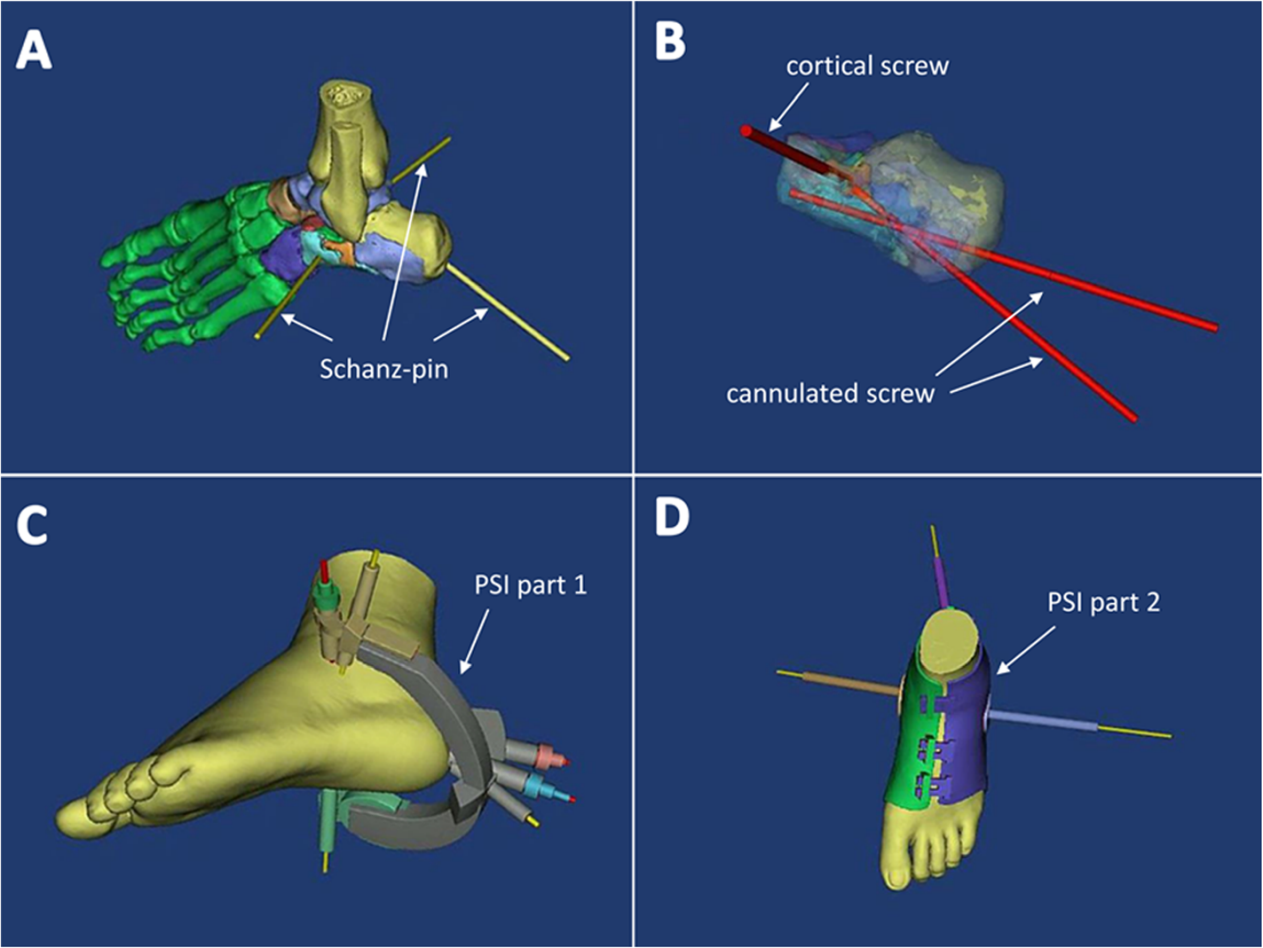
Preoperative planning process. **a** The location of 3 to 5 Schanz pins (or K-wires) for connecting fracture fragments was determined by digital simulation. **b** The number, size, and location of the internal fixation were determined by digital simulation, while ensuring that each Schanz pin (or K-wire) formed a rigid complex with the fracture fragments. **c** PSI part 1, an instrument used to immobilize the calcaneus after reduction and guide the orientation of the screws, was constructed. **d** PSI part 2, which was used to guide the placement of the 3 to 5 Schanz pins (or K-wires), restored the calcaneus to its prefracture state with Schanz pins (or K-wires) connecting the fragments

Preoperative planning was carried out by H.L. and C.G.W. A CT scan (Philips Medical Systems, Eindhoven, Netherlands) of the patient’s foot was obtained at a slice thickness of 0.6 mm, and the image was converted to DICOM format. A 3D model of the calcaneus and skin profile was created with Mimics v19.0 software (Materialise, Leuven, Belgium). The calcaneus was split into a number of fragments (usually 3 to 5). We used the software to mimic anatomic reduction of the calcaneus and generate the PSI. 1) Three to 5 Schanz pins (or K-wires) were used as hand grips to independently connect the fragments that would be used for fracture reduction. 2) Calcaneus fracture reduction was simulated in the digital model in order to decide on the fixation method for the fracture and determine the screw size and orientation. The simulation showed that we needed to insert 1 cortical screw from the outside to the inside of the left foot beneath the posterior subtalar joint, and insert 1 lag screw from the margin of the attachment of the Achilles tendon and calcaneocuboid joint. A second lag screw was required from a point 1–2 cm below the insertion point of the first screw to the middle of the subtalar joint. Part 1 of the PSI reduced and immobilized the calcaneus fracture by means of K-wires passing through and guiding screw orientation. The original state of the calcaneus was restored with Schanz pins connected to the fragments, and part 2 of the PSI marked the position of the Schanz pins (or K-wires). The CT data revealed not only the bone, but also the soft tissue, including the skin profile; the internal profile of PSI part 2 was based on the skin profile of the area to be operated (Fig. 1). Finally, a PSI was 3D-printed based on the digital information by selective laser sintering using nylon as the material.

### Surgery

**Fig. 2:**
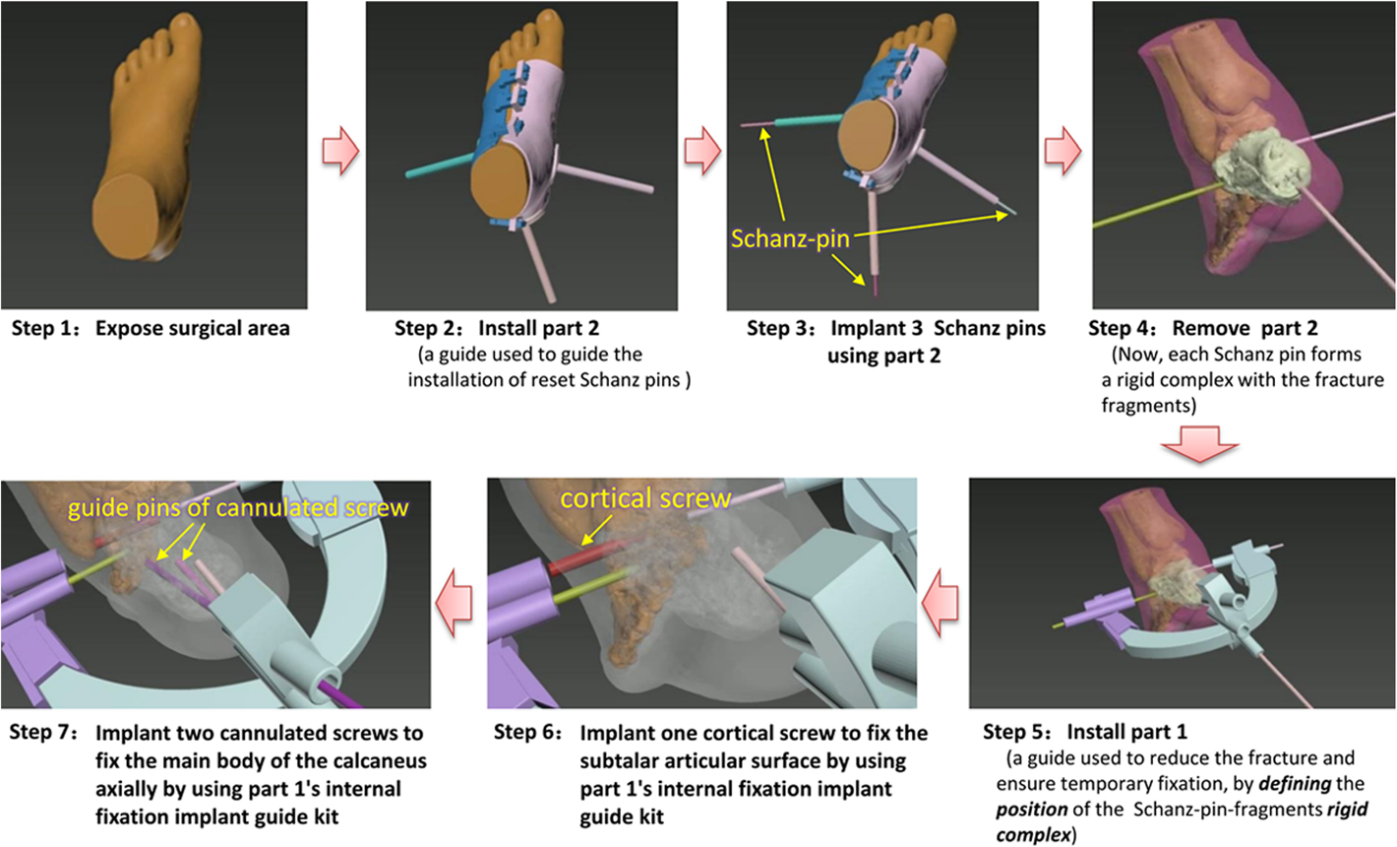
Schematic of the new surgery method. The operation included 7 steps and was PSI-assisted throughout

In a representative case, the patient lay in the supine position under nerve-blocking anesthesia, and a tourniquet was applied to the upper thigh. The surgical procedure was the reverse of the PSI design process, and consisted of the following 7 steps (Fig. 2).

**Fig. 3:**
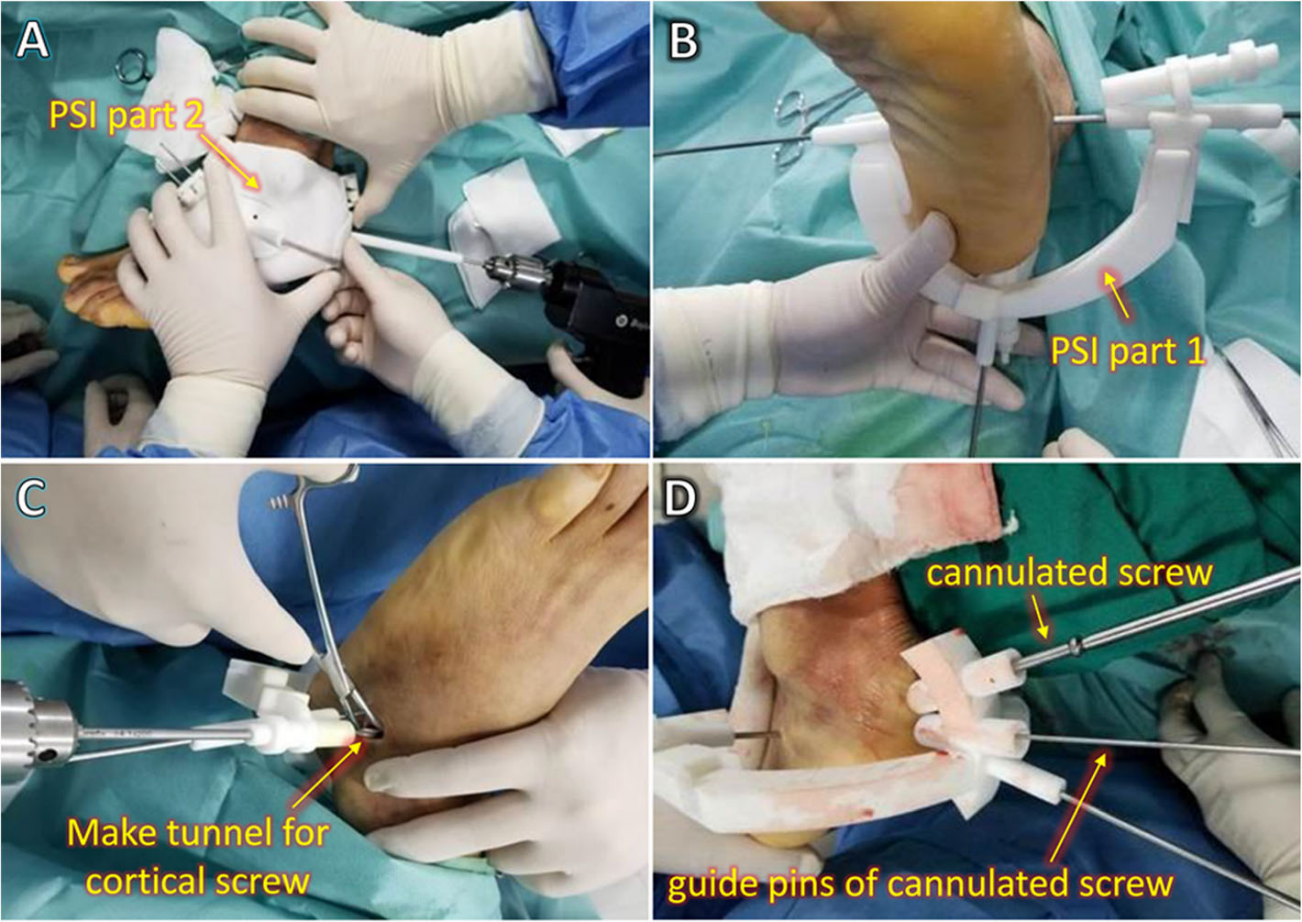
Application of the PSI during surgery. **a** PSI part 1 was assembled, and 3 Schanz pins were used to connect the calcaneus fragments. **b** Anatomic reduction was achieved with guidance by PSI part 2. **c** With guidance by PSI part 2, a lateral screw was inserted to fix the subtalar articular surface. **d** Two posterior cannulated screws were inserted to fix the main body of the calcaneus axially

1. We exposed surgical area.
2. We installed part 2 of the PSI to the left foot and ankle to guide the installation of reset Schanz pins (or K-wires).
3. We inserted 3 to 5 Schanz pins (or K-wires) to independently connect the fragments through the Schanz pin holes of PSI part 2 (Fig. 3**-A**).
4. After removing PSI part 2 with Schanz pins connected to the calcaneus, we were ready to reduce the calcaneus. At this point, each Schanz pin (or K-wire) formed a rigid complex with the fracture fragments.
5. We grasped the Schanz pins (or K-wires) to preliminarily reduce the calcaneus based on our understanding of the characteristics of the displaced fracture. At the same time, PSI part 1 was assembled by passing Schanz pins (or K-wires) through the Schanz pin (or K-wire) holes (Fig. 3**-B**). Ideal anatomic reduction was achieved when PSI part 1 was fixed to the foot with Schanz pins (or K-wires) pushed through the Schanz pin (or K-wire) holes, because the function of part 1 was to delineate the position of the rigid Schanz-pin (K-wire)–fragment complex.
6. We made a 8- to 20-mm–wide incision on the lateral surface of subtalar joint, and implanted 1 or 2 cortical screws to fix the subtalar articular surface using the internal fixation implant guide kit of part 1 (Fig. 3**-C**).
7. We implanted 2 or 3 cannulated screws to fix the main body of the calcaneus axially using the internal fixation implant guide kit of part 1 (Fig. 3**-D**).

After installing all internal fixations, we performed a C-arm check, then disassembled the PSI and removed the Schanz pins (or K-wires).

### Postoperative management

After surgery, patients were required to keep their limb raised and were given antibiotics. The patients wore a plaster slab until the stitches of their wound were removed immediately after surgery, after which they wore a supramalleolar protective plaster cast. Patients were encouraged to engage in non–weight-bearing mobilization for the first 3–8 weeks, followed by partial weight bearing for several weeks according to the doctor’s advice. Full weight bearing was allowed when radiographs showed evidence of osseous union, generally at 3 months post surgery [17, 18].

### Measurement and follow-up

**Fig. 4:**
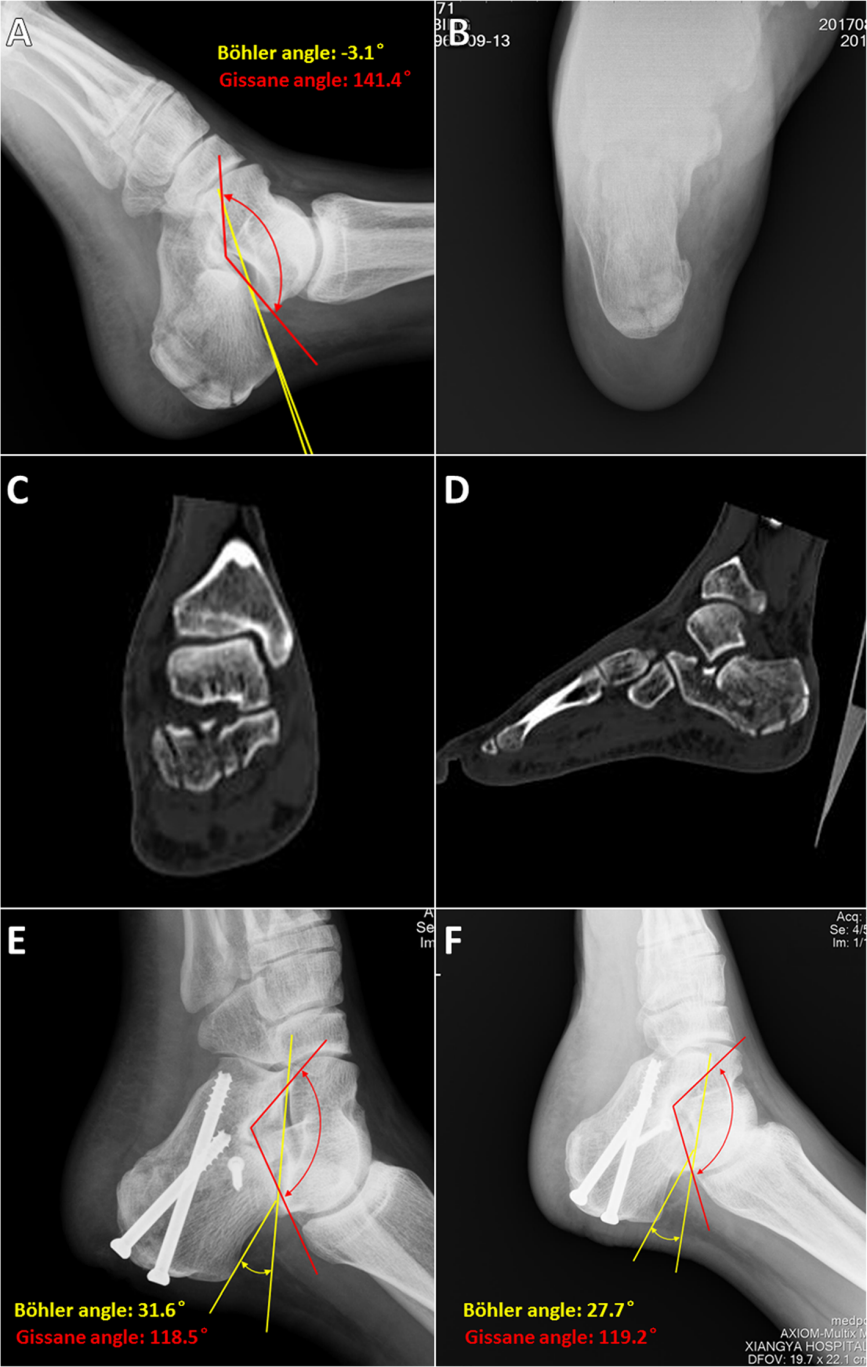
Imaging examination and evaluation of a representative case (male, 47 years old, left Sanders type IV fresh calcaneal fracture). **a** Example of an intra-articular fracture of the calcaneus. Böhler angle was − 3.1° and Gissane angle was 141.4°. **b** Axial view showing a varus deformity of the calcaneus. **c**, **d** Coronal and sagittal views of the calcaneus in the CT scan show an irregular facet of the subtalar joint (Sanders type IV calcaneus fracture). **e** Lateral radiograph obtained 2 days after the surgery confirmed anatomic reduction of the calcaneus and stable fixation. Böhler angle was 31.6° and Gissane angle was 118.5°. **f** Lateral radiograph obtained at approximately 12 months confirmed anatomic reduction and stable fixation, with a Böhler angle of 27.7° and Gissane angle of 119.2°

**Fig. 5:**
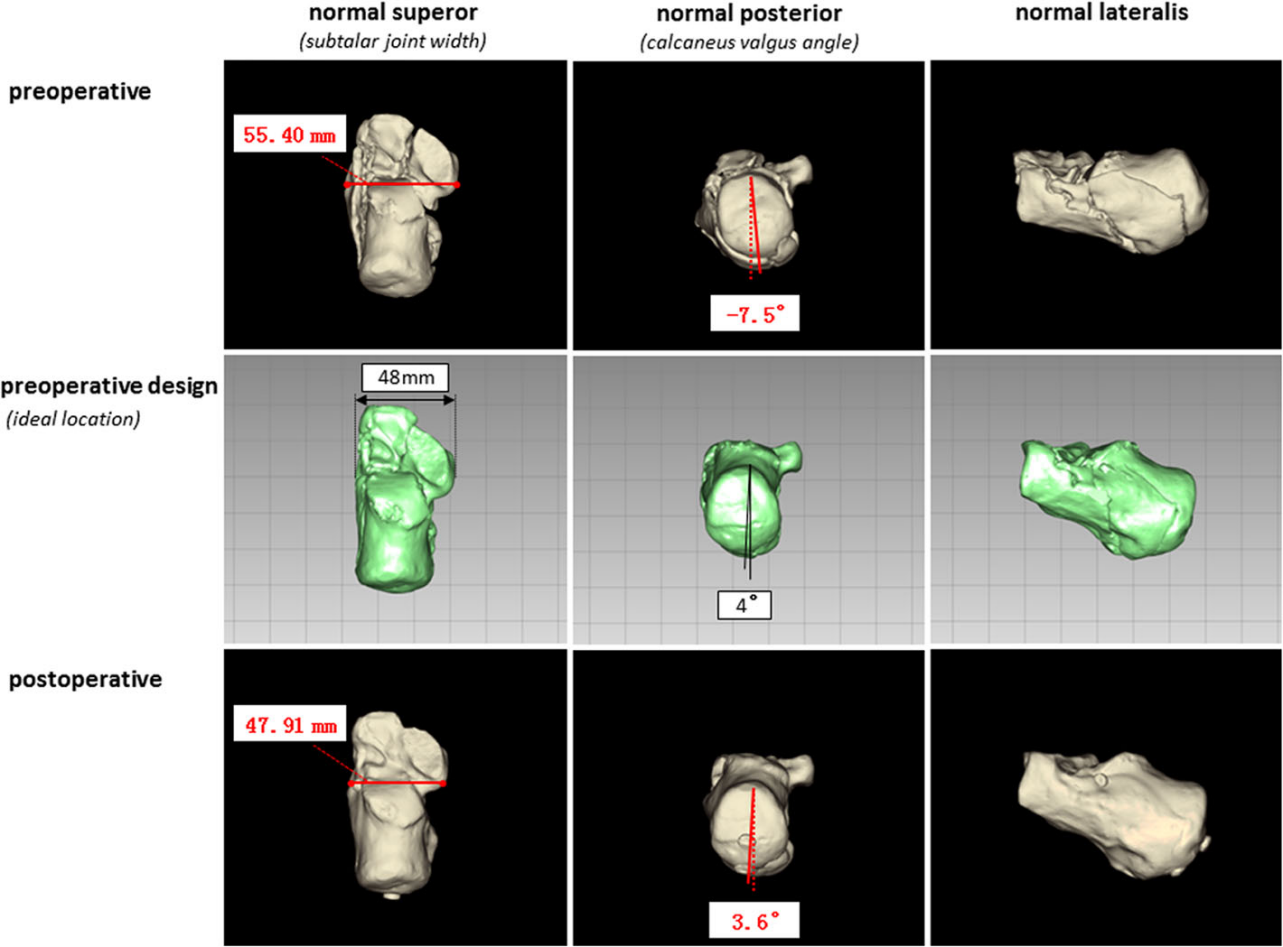
3D Reconstructed model generated based on the preoperative plan and pre- and postoperative actual measurements from CT data. We measured subtalar joint width at the position of the sustentaculum from the normal superior perspective, and the calcaneus valgus angle from the normal posterior perspective

**Fig. 6:**
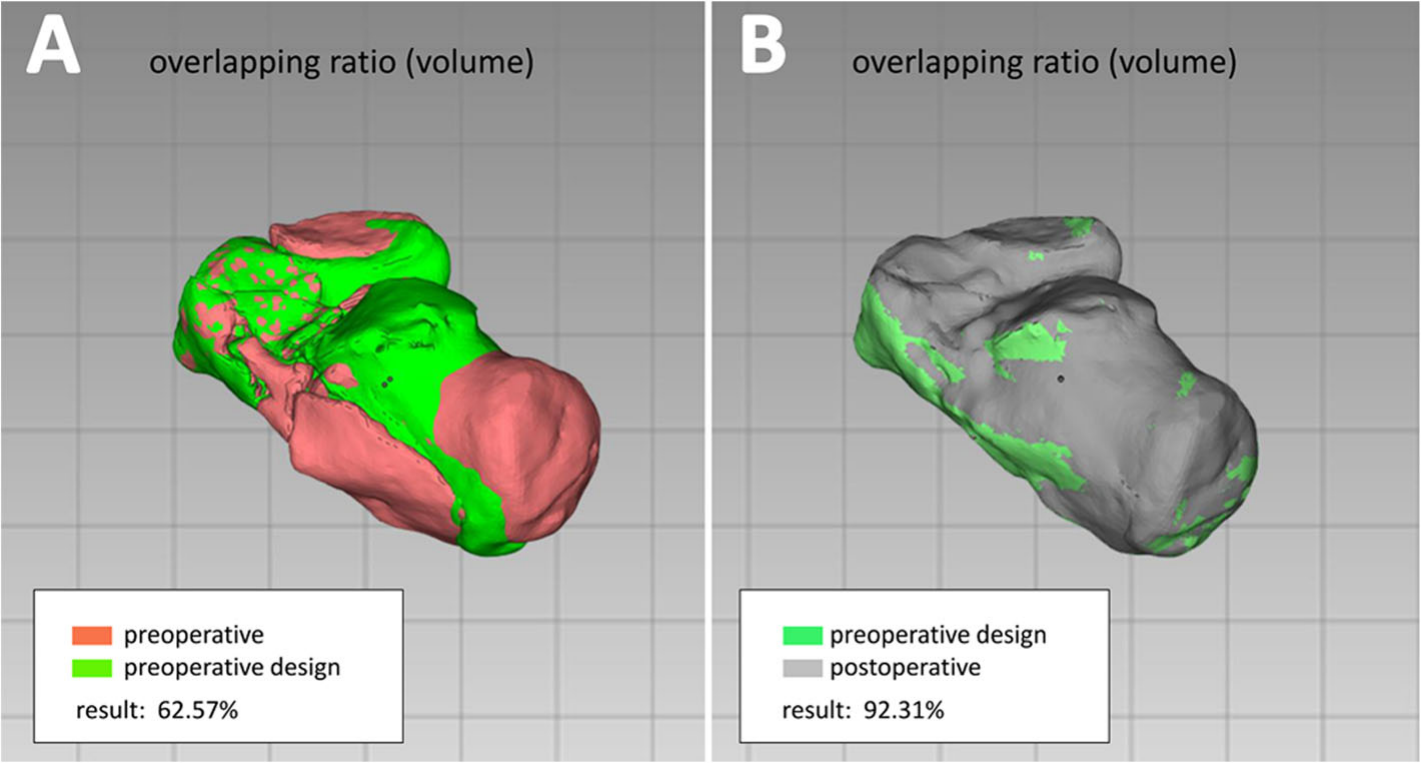
Calcaneal volume overlap ratio. **a** Actual preoperative calcaneal volume overlap ratio compared to the preoperative plan. **b** Calcaneal volume overlap ratio of the preoperative plan compared to postoperative value in which internal fixation has been excluded

We collected the following data from medical records and the follow-up system: intraoperative fluoroscopy time; concurrence between internal fixation actual usage (IFAU) and the preoperative plan, and surgery time and complications. We then gathered information on the preoperative plan and obtained preoperative as well as postoperative actual measurements from standard radiologic examinations (X-ray radiography or CT). We recorded Böhler and Gissane angles from X-ray radiographs (6–12 months after surgery) (Fig. 4). The postoperative CT data (6–12 months after surgery) were transferred to a computer and reconstructed into a postoperative 3D digital model that was compared with preoperative data. We focused on calcaneal shape and anatomic structure, and recorded subtalar joint width (at the position of the sustentaculum) and calcaneus valgus angle (Fig. 5). We overlapped the postoperative 3D calcaneal model with the ideal calcaneal model designed before the surgery and calculated the calcaneal volume overlap ratio (after excluding internal fixation) (Fig. 6). All patients had been followed up, and function score (i.e., American Orthopedic Foot and Ankle Society [AOFAS] score) [19, 20] was recorded 12 weeks, 6 months, and 1 year after surgery. We obtained these data through outpatient or visiting services.

### Statistical analysis

Data are presented as means with ranges. The paired samples t test was used to analyze the preoperative plan and pre- and postoperative data. Statistical analyses were performed using SPSS v22.0 software (SPSS, Chicago, IL, USA). *P* values < 0.05 were considered statistically significant.

## Results

One patient underwent bilateral surgery. The mean intraoperative fluoroscopy time was 3.95 ± 1.78 h (range, 2–9 h). The condition of IFAU in 17 ft (16 patients) was the same as preoperative plan, but in 3 ft (3 patients) there was a discrepancy. The mean surgery time was 28.16 ± 10.70 min (range, 15–50 min). No patient developed complications (Table 2).

**Table 2:** Surgery conditions and complications

Parameter	Value
Intraoperative fluoroscopy (number of times)	3.95 ± 1.78
IFAU same as preoperative plan? (yes/no)	16/3
Surgery time (min)	28.16 ± 10.70
Complications	None

*IFAU* internal fixation actual usag

**Fig. 7:**
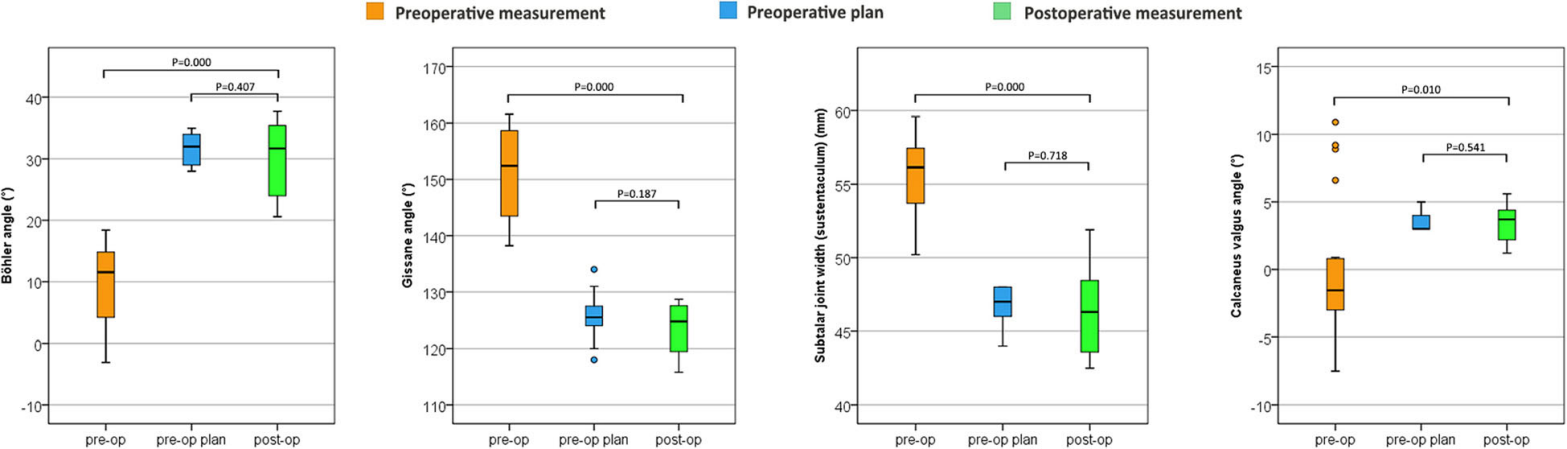
Box diagrams of Böhler, Gissane, and calcaneus valgus angles and subtalar joint width. Postoperative measurements did not differ significantly from the preoperative plan, but significant differences were observed between pre- and postoperative actual measurements

The Böhler angle in the preoperative plan was 31.5° ± 2.7° (range, 28°–35°), and the postoperative angle was 30.2° ± 5.9° (range, 20.6°–37.7°); the difference (5.7° ± 3.3°; range, 0.7°–12.2°) was not statistically significant (*P* = 0.407). However, the actual measured preoperative Böhler angle was 9.6° ± 6.5° (range, − 3.1°–18.4°), which differed significantly from the postoperative Böhler angle of 20.7° ± 7.0° (range, 9.3°–36.8°) (*P* = 0.000). The Gissane angle in the preoperative plan was 125.6° ± 4.0° (range, 118°–134°), and the postoperative angle was 123.6° ± 4.4° (range, 115.8°–128.7°); the difference between the 2 values (5.0° ± 4.3°; range, 1.0°–17.0°) was non-significant (*P* = 0.187). However, the actual measured preoperative Gissane angle was 151.3° ± 8.0° (range, 138.2°–161.5°), which was significantly different (*P* = 0.000) from the postoperative Gissane angle of 27.7° ± 7.7° (range, 14.0°–41.5°). The preoperative plan subtalar joint width (at the position of the sustentaculum) was 46.8 ± 1.3 mm (range, 44–48 mm), while the postoperative subtalar joint width was 46.4 ± 2.9 mm (range, 42.5–51.9 mm); the difference between the values (3.2 ± 2.0 mm; range, 0.1–7.9 mm) was nonsignificant (*P* = 0.718). However, the actual measured preoperative subtalar joint width was 55.4 ± 2.8 mm (range, 50.2–59.6 mm), which differed from the postoperative value of 9.0 ± 4.3 mm (range, 2.4–16.4 mm) (P = 0.000). The preoperative plan calcaneus valgus angle was 3.6° ± 0.8° (range, 3°–5°), and the postoperative angle was 3.3° ± 1.3° (range, 1.2°–5.6°); the difference of 1.4° ± 0.9° (range, 0.3°–3.6°) between the values was not statistically significant (*P* = 0.541). However, the actual measured preoperative calcaneus valgus angle was − 0.1° ± 5.0° (range, − 7.5°–10.9°), which differed (*P* = 0.010) from the postoperative angle of 4.6° ± 1.9° (range, − 5.3°–8.7°). The calcaneal volume overlap ratio in the preoperative design was 91.2 ± 2.3% (range, 87.0%–95.8%) (Table 3 and Fig. 7).

**Table 3:** Comparison of preoperative plan vs postoperative actual measurements and pre- vs postoperative actual measurements based on X-ray radiography and CT 3-dimensional reconstruction data

Mean		Preoperative plan vs postoperative actual measurement				Preoperative vs postoperative actual measurement			
		Preoperative plan	Postoperative	Δ^b^	P^a^	Preoperative	Postoperative	Δ^b^	P^a^
X-ray	Böhler angle (°)	31.5 ± 2.7	30.2 ± 5.9	5.7 ± 3.3	0.407	9.6 ± 6.5	30.2 ± 5.9	20.7 ± 7.0	0.000
	Gissane angle (°)	125.6 ± 4.0	123.6 ± 4.4	5.0 ± 4.3	0.187	151.3 ± 8.0	123.6 ± 4.4	27.7 ± 7.7	0.000
CT 3-dimensional reconstruction	Subtalar joint width (sustentaculum) (mm)	46.8 ± 1.3	46.4 ± 2.9	3.2 ± 2.0	0.718	55.4 ± 2.8	46.4 ± 2.9	9.0 ± 4.3	0.000
	Calcaneus valgus angle (°)	3.6 ± 0.8	3.3 ± 1.3	1.4 ± 0.9	0.541	−0.1 ± 5.0	3.3 ± 1.3	4.6 ± 1.9	0.010
	Calcaneal volume overlap ratio with preoperative design:	91.2 ± 2.3%							

^a^Paired-samples t test

^b^Difference in absolute value

**Fig. 8:**
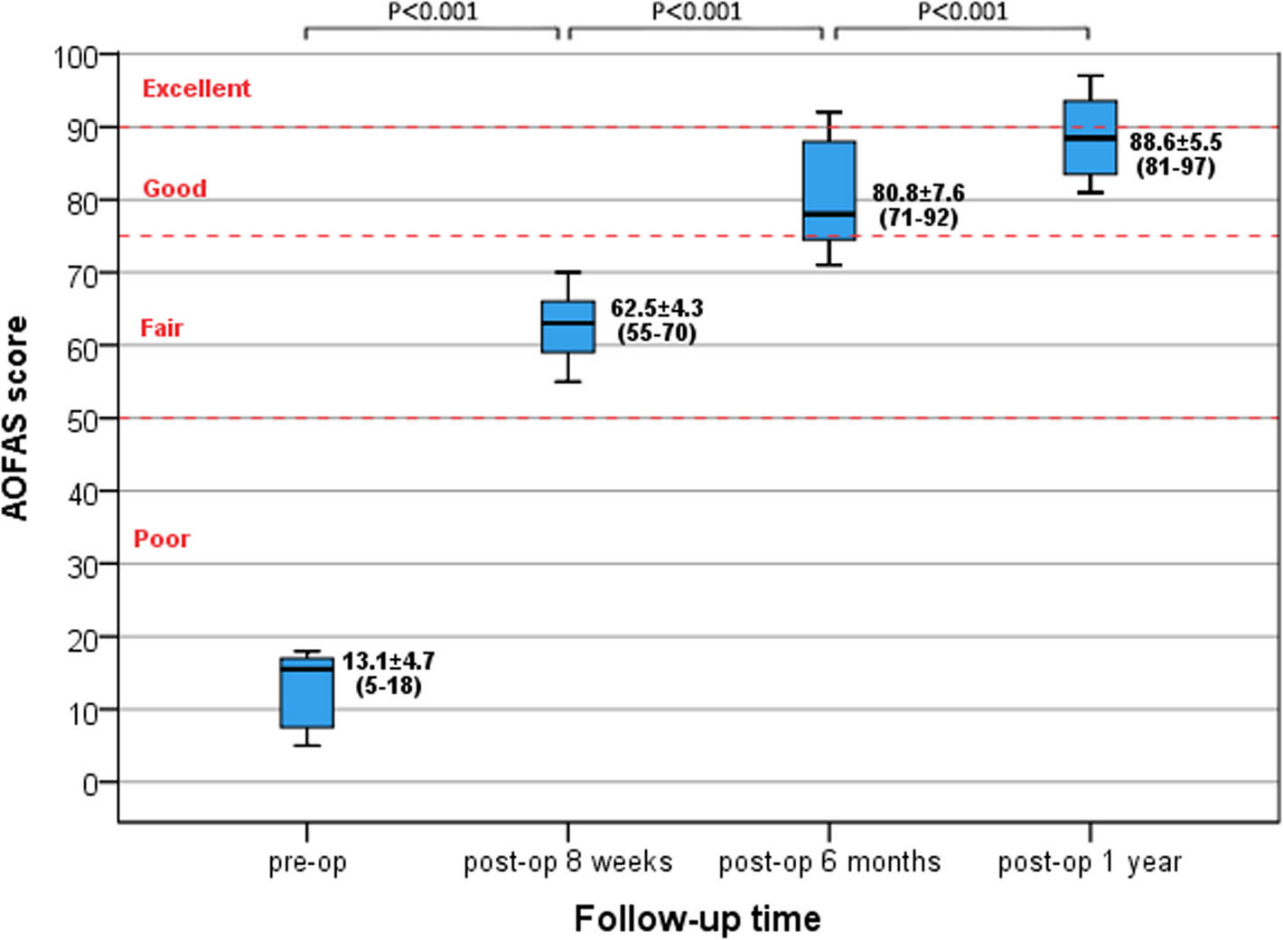
Box diagrams of the AOFAS score of patients at each time point. AOFAS scores increased with time, and differences in scores at each time point were significant

All patients had 3 postoperative follow-up examinations; the AOFAS score of all patients increased with time. The preoperative AOFAS score was 13.1 ± 4.7 (range, 5 to 18); and postoperative scores were as follows: 8 weeks, 62.5 ± 4.3 (range, 55–70); 6 months, 80.8 ± 7.6 (range, 71–92); and 1 year, 88.6 ± 5.5 (range, 81–97). The differences in AOFAS scores at each time point were statistically significant (*P* < 0.001) (Fig. 8).

## Discussion

For decades, open reduction and internal fixation performed using an extended extensile lateral approach has been the standard treatment for calcaneal fracture [21]. This method achieves a certain degree of anatomic reduction, but the occurrence of serious complications has prompted the development of less invasive approaches [22]. A minimally invasive sinus tarsi approach for anatomic reduction and stable fixation of complex calcaneal fractures has been described [23]; and in a study of 156 patients, percutaneous leverage, manual compression, and application of anatomic plates and compression bolts applied using a minimally invasive lateral approach were effective in the treatment of displaced intra-articular calcaneal fractures, with fewer soft tissue complications and good fracture reduction [24]. However, the classic minimally invasive surgical method for calcaneal fractures—which involves fracture reduction, temporary fixation, and internal fixation with an implant—has certain disadvantages. Moreover, most surgical procedures require extensive intraoperative fluoroscopy, and the successful execution of each step depends on the surgeon’s experience, which can lead to suboptimal outcomes.

The approach used in minimally invasive surgery for calcaneus fracture is determined by the fracture pattern; thus, personalized surgical planning is critical [25]. By CT imaging and use of a prototype produced rapidly by 3D printing, the surgeon can obtain detailed information on the fracture and plan a procedure that will yield satisfactory fixation [26]. More importantly, the surgery can be simulated in vitro. Preoperative planning tends to be idealized and it is not possible to fully anticipate the surgical challenges in individual cases. Calcaneus fractures are complex [27], and there is no single treatment protocol that is suitable for all of the different types. The main point of the PSI was to guide the surgery according to the plan. We used a computer-generated model of the calcaneus in order to examine the features of the fracture [28] and simulate the reduction and fixation, then created a PSI to guide the actual surgery.

We set out to optimize the classic method of calcaneal fracture MIIF by making it more personalized and precise. To this end, we designed a surgical procedure based on a simulation and then performed PSI-assisted operation. The unique aspect of the present case is that the traditional calcaneal fracture MIIF procedure was modified so that it consisted of preoperative digital surgical simulation and preparation of the PSI, installation of Schanz pins (or K-wires) using the PSI, adjustment of the relationship between Schanz pins (or K-wires) and the PSI according to the surgical plan, and internal fixation with an implant using the PSI. Thus, the focus of the surgical procedure changed from internal conditions to external auxiliary tools, resulting in an operation that was better planned and more rapidly and accurately executed.

In this work we not only described a new type of PSI, but also demonstrated a novel method for internal fixation of calcaneal fractures. In other words, the whole surgery was PSI-assisted and the preoperative plan was executed step-by-step, which is fundamentally different from traditional surgery in which each step is improvised and is associated with a degree of uncertainty. In contrast, the procedure described in the present study is standardized and methodical and the whole operation can be improved or even modified, unlike the traditional PSI method in which only part of the procedure is optimized. Thus, our newly developed strategy can be used to accurately execute the preoperative plan. Indeed, the actual postoperative measurements of Böhler, Gissane, and calcaneus valgus angles and subtalar joint width (sustentaculum) did not deviate significantly from the preoperative plan, and the postoperative calcaneal volume overlap ratio with the preoperative design was 91.2% ± 2.3%.

The design of the guide plate and in particular, the correct location for installation of PSI part 2, is critical for successful fracture reduction surgery. CT scans not only reveal the bone but also the profile of soft tissues including skin. The internal profile of PSI part 2 was based on the skin profile of the operated area. However, 3 points are worth noting. Firstly, the internal profile of PSI part 2 was slightly larger than the skin profile of the surgical area, and we usually left a ~ 1-mm gap between them. Secondly, in order to minimize swelling in the surgical area and the effect on the skin profile, we improved PSI part 2 as follows: to accommodate any swelling, we left a larger gap in the area that did not correspond to the superficial bony marks at the closest and furthest ends of PSI part 2; and we designed PSI part 2 as 2 pieces of shell-like armor connected by a lock in a sliding slot at a limited distance that could accommodate soft tissue swelling. It is important to note that the distance of the slot prevented the 2 pieces of armor from shifting across the cross-section; in repeated computer simulations, this small movement did not affect the position of the Schanz pins (or K-wires). Thirdly, before the operation, we strictly advised the patient to adopt measures to minimize limb swelling such as raising the affected limb, using an ice compress, braking, and taking anti-inflammatory drugs.

The new operation method was simple and the surgery process was smooth, and a good postoperative effect was achieved. Moreover, the surgery time was just 28.16 ± 10.70 min as compared to > 60 min for classic calcaneal fracture MIIF and ORIF [17, 29]. The operation was guided at each step by the PSI, which facilitated the surgical procedure and eliminated the need for extensive intraoperative fluoroscopy, resulting in near-perfect fracture reduction and internal fixation.

### Limitations of PSI-assisted MIIF of calcaneal fracture

The procedure described in this study has some limitations. Firstly, mastering new technology to design a reliable and effective PSI is a complex process that takes time and experience [30, 31]. In the first year of implementation, there were 3 cases (19 in total) in which the IFAU diverged from the preoperative plan. Specifically, the length of the cannulated screws required to fix the main body of the calcaneus axially during the operation deviated from the preoperative plan, which affected fixation of the fracture pieces or the functioning of the subtalar joint. In 2 cases, the screws were too long and penetrated the subtalar joint; and in another case, a screw that was too short resulted in fracture blocks that could not be firmly fixed. We have since performed more detailed tests and made improvements to the simulated preoperative plan; as a result, the situations just described never recurred. In our experience, the process of mastering this new technique takes about 1 year or 5 cases.

Secondly, the technique is not suitable for managing a fracture 72 h after injury. We used only Schanz pins (or K-wires) to reduce a fresh fracture; after 72 h has elapsed, closed reduction of an injury is not possible. Ideally, PSI-assisted surgery should be performed within 8 h after injury. Based on our experience, although PSI part 1 can facilitate the reduction of the fracture block within 72 h of injury, the optimal time to carry out the operation is within 8 h when there is minimal swelling of soft tissue, which is conducive to the installation of PSI part 2. However, it is difficult to carry out this surgical technique within 8 h if the surgeon is not sufficiently skilled in surgical design and 3D printing of the PSI takes a long time.

A third limitation of the present study is that this was a preliminary application of a method in a small sample of patients and short follow-up period. Therefore, prospective investigations with the large sample sizes and longer follow-up times are needed to evaluate the clinical applicability of PSI-assisted MIIF of calcaneal fractures.

## Conclusions

We developed a surgical procedure for calcaneal fracture MIIF that is focused on external auxiliary tools rather than internal conditions as in the traditional method. Our results show that with PSI-assisted calcaneal fracture MIIF the preoperative plan can be accurately and rapidly executed, which can improve surgical outcomes.

## Supplementary information

**Additional file 1.** Video 1: Example of the process of PSI preoperative design and surgical application.

## Data Availability

The datasets used and/or analyzed during the study are available from the corresponding author upon reasonable request.

## Abbreviations

AOFAS: American Orthopedic Foot and Ankle Society
CT: Computed tomography
IFAU: Internal fixation actual usage
MIIF: Minimally invasive internal fixation
ORIF: Open reduction internal fixation
PSI: Patient-specific instrument
